# Exergy Efficiency of Closed and Unsteady-Flow Systems

**DOI:** 10.3390/e27090943

**Published:** 2025-09-10

**Authors:** Yunus A. Çengel, Mehmet Kanoğlu

**Affiliations:** 1 Department of Mechanical Engineering, University of Nevada, Reno, NV 89557, USA; 2 Department of Mechanical Engineering, Alanya Alaaddin Keykubat University, Antalya 07450, Türkiye; mehmet.kanoglu@alanya.edu.tr

**Keywords:** thermodynamics, exergy, exergy analysis, exergy efficiency, exergy destruction, second-law analysis, second-law efficiency

## Abstract

Exergy efficiency is viewed as the degree of approaching reversible operation, with a value of 100 percent for a reversible process characterized by zero entropy generation or equivalently zero exergy destruction since *X*_destroyed_ = *T*_0_*S*_gen_. As such, exergy efficiency becomes a measure of thermodynamic perfection. There are different conceptual definitions of exergy efficiency, the most common ones being (1) the ratio of exergy output to exergy input *η*_ex_ = *X*_output_/*X*_input_ = 1 − (*X*_destroyed_ + *X*_loss_)/*X*_input_, (2) the ratio of the product exergy to fuel exergy *η*_ex_ = *X*_product_/*X*_fuel_ = 1 − (*X*_destroyed_ + *X*_loss_)/*X*_fuel_, and (3) the ratio of exergy recovered to exergy expended *η*_ex_ = *X*_recovered_/*X*_expended_ = 1 − *X*_destroyed_/*X*_expended_. Most exergy efficiency definitions are formulated with steady-flow systems in mind, and they are generally applied to systems in steady operation such as power plants and refrigeration systems whose exergy content remains constant. If these definitions are to be used for closed and unsteady-flow systems, the terms need to be interpreted broadly to account for the exergy change of the systems as exergy input or output, as appropriate. In this paper, general exergy efficiency relations are developed for closed and unsteady-flow systems and their use is demonstrated with applications. Also, the practicality of the use of the term exergy loss *X*_loss_ is questioned, and limitations on the definition *η*_ex_ = *W*_act,out_/*W*_rev,out_ are discussed.

## 1. Introduction

The second-law or exergy analysis of energy systems is usually considered a challenging experience by engineering students in thermodynamics courses as well as practicing engineers due to the abstract nature of the topic. A key parameter of second-law analysis of energy systems is *exergy efficiency*—also known as exergetic efficiency, second-law efficiency, effectiveness, or second-law effectiveness. We prefer the phrase *exergy efficiency* to *exergetic efficiency* since it is simpler and it parallels the commonly used phrase *energy efficiency*. It takes time to develop an intuitive understanding and working knowledge of the second-law concepts of entropy, entropy generation, exergy, exergy destruction, and exergy efficiency. This is partly because, unlike energy, entropy is generated, and exergy is destroyed rather than being conserved. As a result, there is a tendency to do away with exergy balance and exergy efficiency, and limit thermodynamic analysis to energy balance and energy conversion efficiencies.

The performance of heat engines, for example, is usually assessed by first-law or thermal efficiency, which represents the fraction of heat input converted to work. But thermal efficiency does not give the full picture of performance. A thermal efficiency of 40 percent may appear low for a power plant that absorbs heat from a source at 900 K and rejects the waste heat to the environment at 300 K. But the upper limit for the thermal efficiency for this power plant, based on ideal Carnot cycle operation, is 1 − 300/900 = 0.67 or 67 percent rather than 100 percent, and thus the 40 percent actual thermal efficiency does not look so bad when compared to 67 percent rather than 100 percent. The ratio of actual thermal efficiency to the maximum (Carnot) efficiency is the *exergy efficiency* of this plant, which is 40/67 = 0.60 or 60 percent. Unlike Carnot efficiency, the upper limit of exergy efficiency is 100 percent. As such, exergy efficiency is always a good indicator of how well an energy system or device is performing compared to the best possible performance.

Second-law or exergy analysis is often viewed as a topic of academic interest rather than a practical tool. One reason for this perception is the inadequate coverage of exergy efficiency in thermodynamics and thermal sciences textbooks [1,2,3,4]. Exergy efficiency coverage is usually limited to common steady-flow devices such as turbines, compressors, pumps, heat exchangers, and mixing chambers. There is hardly any coverage of the *exergy efficiency* of closed systems such as rigid tanks and piston–cylinder devices and unsteady-flow processes. This is also the case for the books that focus on the exergy concept and provide extended coverage of exergy analysis and its applications [5,6,7,8].

There are so many studies in the literature on energy systems that make use of exergy efficiency as part of the analysis. For example, Paul et al. [9] performed exergy characterization of a compression–ignition (CI) engine and calculated exergy efficiency using the relation *η*_ex_ = (Shaft power)/(Inlet air exergy + Chemical exergy of fuel). Chen et al. [10] evaluated a modified ejector-enhanced refrigeration cycle using energy and exergy analysis and determined exergy efficiency from *η*_ex_ = 1 − (Total exergy destroyed)/(Compressor power). Yu et al. [11] used *η*_ex_ = (Exergy output)/(Exergy input) to calculate the exergy efficiencies of all the components of a trigeneration system for power, heat, and cooling. Ibrahim et al. [12] used the relation *η*_ex_ = 1 − (Exergy destroyed)/(Compressor power input + Exergy at compressor inlet) to evaluate compressor exergy efficiency and the relation *η*_ex_ = 1 − (Exergy destroyed)/(Turbine power output + Exergy at turbine inlet) to calculate turbine exergy efficiency in their analysis of a gas turbine power plant. These relations for exergy efficiency are not consistent with the relations used by most other researchers for the same components. Fu et al. [13] analyzed a gasoline engine and used the relation *η*_ex_ = (Exergy of heat energy)/(Fuel chemical energy) to determine the exergy efficiency of the engine. It is noticed that the authors used an energy term in the denominator rather than exergy.

Onigbajumo et al. [14] evaluated the exergy efficiency of a solar-driven water gasification process as the ratio of exergy available in the final product stream to the total exergy absorbed in the inlet stream. Abedin and Rosen [15] presented the exergy efficiency of a charging process as the exergy accumulation over the exergy input and that of thermochemical storage as recovered exergy over exergy input. Gurbuz et al. [16] calculated exergy efficiency of a diffusion absorption refrigeration system using the expression *η*_ex_ = 1 − (Total exergy destroyed)/(Exergy input), but it was not clear what exactly the exergy input represented in the system. Molina-Salas et al. [17] used two different relations to calculate the exergy efficiency of a simple off-shore oscillating water column device: *η*_ex_ = (Exergy output)/(Exergy input) and *η*_ex_ = 1 − (Exergy destruction at products)/(Exergy destruction at sources). These two relations do not seem to be consistent with each other. Kanoglu [18] used two different exergy efficiency relations when evaluating the performance of a geothermal power plant. Kanoglu [19] developed exergy efficiency relations for a multistage cascade refrigeration cycle used for natural gas liquefaction. Brötz et al. [20] defined the exergy efficiency of a fan as the ratio of exergy change of the fluid across the fan to the shaft power input.

The performance of various energy systems is analyzed and optimized using second-law analysis which includes exergy efficiency. Some examples in the literature include combined cycle power plants [21], regenerative closed Brayton cycles [22], reversible absorption heat pumps [23], dual-pressure organic Rankine cycles [24], integrated natural gas liquids and liquefied natural gas processes [25], drying plants [26], steam power plant [27], ground-source heat pumps [28], a methanol production plant [29], ecologically regenerative building systems [30], and industrial pneumatic systems [31].

There exist different viewpoints on how exergy efficiency should be defined, and applying a particular definition zealously to all processes, devices, or systems is common. This practice often results in inconsistencies and confusion about exergy efficiency. Consequently, there exist different definitions of exergy efficiency, and thus the exergy efficiency of a process can be different, depending on which definition is used. Here, the challenge is to pick the definition that is most suitable for the situation at hand by considering the desired output of the installed device or system and the resources consumed. In this paper, general definitions of exergy efficiencies are developed for closed and unsteady-flow systems, and their use is demonstrated by applying them to specific systems. But first, a general overview of exergy and chemical exergy is given to serve as the proper background, especially for those who are not up to date with exergy analysis for easy reference.

## 2. An Overview of Exergy

Exergy is the work potential of energy. It is the *maximum useful work* a system at a specified state could produce in a given environment, which occurs when the system undergoes a totally reversible process from its given state to the state of the environment. Alternately, exergy is the *minimum useful work required* to bring a system from the state of the environment to a specified state, which occurs when the system undergoes a totally reversible process from the state of the environment to the specified state. Therefore, exergy is a measure of departure of the state of the system from the state of the environment, and no useful work can be produced from a system that is in the state of its environment.

Exergy is a property of the system and environment combined. But for an environment of fixed properties, exergy becomes the property of the system. Energy is conserved, but exergy is not—it can be destroyed. Exergy destruction is zero and thus exergy remains constant only in the limiting case of a totally reversible process—a process that involves no irreversibilities such as friction, heat transfer through a finite temperature difference, unrestrained expansion, mixing, and chemical reactions.

The region of space a system operates in is referred to as the *environment*. Everything outside the system boundaries constitutes the *surroundings* of the system. The part of the surroundings whose properties are affected by the interactions between the system and the environment is called the *immediate surroundings*. In exergy analysis, the environment is idealized as a large simple compressible system at a *uniform temperature* of *T*_0_, *pressure* of *P*_0_, and a fixed chemical composition of *i* components with *chemical potential μ*_0,*i*_ in the standard state. The environment *excludes* the region of immediate surroundings where gradients occur and properties deviate from the fixed values of *T*_0_, *P*_0_, and *μ*_0,*i*_. As such, the environment is free of irreversibilities and its intensive properties such as temperature, pressure, and chemical composition are not affected by the heat, work, and mass interactions with the system. The environment is also considered to be *at rest* and thus to have no kinetic or potential energy. Irreversibilities that occur within the system constitute *internal irreversibilities*, and those that occur within the immediate surroundings constitute *external irreversibilities*. The sum of the two gives the *total irreversibilities*.

A system is said to be in a *dead state* when it is in thermal, mechanical, and chemical equilibrium with its environment and thus has the same temperature *T*_0_, pressure *P*_0_, and chemical potentials *μ*_0,*i*_ of the environment. The *standard environment* is usually characterized by *T*_0_ = 25 °C, *P*_0_ = 1 atm, and a set of reference substances with *standard concentrations* that closely resemble the chemical makeup of the natural environment.

The system in the dead state also has *zero velocity* and *zero elevation* relative to the environment, and no work can be extracted from it. If the system is *impervious* to mass flow, the chemical equilibrium between the system and the environment is irrelevant, and having thermal and mechanical equilibrium with the environment will suffice. Knowledge of the chemical composition of the environment is not necessary in this case. A system in thermal and mechanical equilibrium with its environment and thus at the same temperature *T*_0_ and pressure *P*_0_ is said to be in the *restricted dead state*.

*Energy* can exist in many forms such as thermal, mechanical, kinetic, potential, electric, magnetic, chemical, and nuclear. There is *exergy* (work potential) associated with each form of energy, and the sum of the exergies associated with each relevant form of energy gives the *total exergy X* (or *x* on a unit mass basis) of the system. The sum of all forms of energy related to the microscopic structure of a system and the degree of molecular activity, such as thermal, chemical, and nuclear energies, is called *internal energy U* (or *u* on a unit mass basis). The sensible and latent forms of internal energy are known as *thermal energy*, and internal energy can be viewed as the sum of *thermal*, *chemical,* and *nuclear* energy. The combination of internal energy *u* and *flow energy Pv* (or *P*/*ρ*) associated with a flow stream is *enthalpy h* = *u* + *Pv*. Kinetic energy ke = *V*^2^/2 and potential energy pe = *gh* are *macroscopic* forms of energy that a system possesses with respect to some outside reference frame. Here, *V* is the velocity of the system relative to the environment, *g* is the gravitational acceleration, and *z* is the elevation of the system relative to a reference level in the environment. For rotating bodies, kinetic energy also includes *rotational* kinetic energy.

Kinetic and potential energies can be converted completely to mechanical work, and thus they are properly categorized as *mechanical energy*. This is also the case for flow energy *e*_pv_, except that the work carried out against surroundings *P*_0_*v* should be subtracted to obtain the *useful work* and thus the exergy. The exergy *x* associated with mechanical forms of energy can be expressed as follows:

*x*_ke_ = ke = *V*^2^/2 (1)

*x*_pe_ = pe = *gz* (2)

*x*_pv_ = *e*_pv_ − *e*_p0v_ = (*P − P*_0_)*v* = (*P − P*_0_)/*ρ* (3)

In the absence of electric, magnetic, and surface tension effects, the total energy of a system made up of *nonflowing mass* consists of internal energy *u* (which is the sum of thermal, chemical, and nuclear energies, *u* = *e*_th_ + *e*_ch_ + *e*_nc_), kinetic energy ke, and potential energy pe. The energy of a system made up of a *flowing mass* is obtained by adding flow energy *Pv* to the energy of a nonflowing mass, *e*_flow_ = *e*_nonflow_ + *e*_pv_ = *e*_nonflow_ + *Pv*:

*e*_nonflow_ = (*e*_th_ + *e*_ch_) + ke + pe = *u* + ke + pe = *u* + *V*^2^/2 + *gz* (4)

*e*_flow_ = *e*_nonflow_ + *e*_pv_ = *u* + ke + pe + *Pv* = *h* + ke + pe = *h* + *V*^2^/2 + *gz* (5)

The corresponding *exergy* expressions are obtained by undertaking a reversible process from the given state (no subscript) to the state of the environment (the dead state with ‘0’ subscript) [1], with the following results:

*x*_nonflow_ = (*u − u*_0_) + *P*_0_(*v − v*_0_) − *T*_0_(*s − s*_0_) + ke + pe (6)

*x*_flow_ = (*h − h*_0_) − *T*_0_(*s − s*_0_) + ke + pe (7)

where *u*_0_, *v*_0_, *s*_0_, and *h*_0_ are the internal energy, specific volume, entropy, and enthalpy of the system *in the state of the environment*, respectively. Note that the thermal exergy of the flowing or nonflowing mass is zero at the environmental temperature and pressure, and thus *x*_nonflow_ and *x*_flow_ expressions represent the *useful work potential* (i.e., *exergy*) of the system in the given state per unit mass. The *total exergies* of a nonflow system of mass *m*_1_ in state 1 and a fluid of mass *m*_2_ entering or exiting the system in state 2 are expressed as follows:

(8) $$X_{\mathrm{sys},1}=X_{\mathrm{nonflow},1}=m_{1}x_{\mathrm{nonflow},1}=m_{1}[\left(u_{1}-u_{0}\right)+P_{0}\left(v_{1}-v_{0}\right)-T_{0}\left(s_{1}-s_{0}\right)+{\mathrm{ke}}_{1}+{\mathrm{pe}}_{1}]$$

(9) $$X_{\mathrm{mass},2}=X_{\mathrm{flow},2}=m_{2}x_{\mathrm{flow},2}=m_{2}[\left(h_{2}-h_{0}\right)-T_{0}\left(s_{2}-s_{0}\right)+{\mathrm{ke}}_{2}+{\mathrm{pe}}_{2}]$$

For a system that is *at rest* relative to the environment, exergy terms associated with kinetic and potential energies are zero, ke_1_ = pe_1_ = 0. When there are no chemical or nuclear reactions, the *composition* of the system at rest remains unchanged during the process from state 1 at *T*_1_ and *P*_1_ to the restricted dead state 0 at *T*_0_ and *P*_0_, and the two exergy relations above represent the *thermal exergy* of the nonflowing system and the *thermal* plus *flow exergy* of the flow system.

There is additional useful work potential associated with the process from *the restricted dead state* to the *dead state* so that the system reaches chemical equilibrium with the environment, and it is called *chemical exergy*. This process is usually associated with mixing, and the process does not need to involve chemical reactions. Most systems do not involve mixing, the separation of a mixture into its constituents, chemical reactions, or nuclear reactions, and knowledge of thermal exergy values in relevant states is sufficient in the exergy analysis. This is because chemical and nuclear exergy values remain the same in all states and they cancel each other out when exergy changes are evaluated.

When a flow or nonflow system of mass *m* undergoes a process from state 1 to state 2, the exergy change of the system is the difference between the exergies of the system at the final and initial states:

Δ*X*_nonflow_ = *m*(*x*_nonflow, 2_ − *x*_nonflow, 1_) = *m*[(*u*_2_
*− u*_1_) + *P*_0_(*v*_2_
*− v*_1_) − *T*_0_(*s*_2_
*− s*_1_) + (ke_2_ − ke_1_) + (pe_2_ − pe_1_)] (10)

Δ*X*_flow_ = *X*_2_
*− X*_1_ = *m*(*x*_flow,2_ − *x*_flow,1_) = *m*[(*h*_2_
*− h*_1_) − *T*_0_(*s*_2_
*− s*_1_) + (ke_2_ − ke_1_) + (pe_2_ − pe_1_)] (11)

Exergy is *transferred* to or from a system via three mechanisms: *heat*, *work,* and *mass transfer*. Exergy transfer by mass *m* crossing the system boundary in state 1 is expressed in terms of the exergy of fluid per unit mass *x*_flow_ as follows:

(12) $$X_{\mathrm{mass},1}=X_{\mathrm{flow},1}=mx_{\mathrm{flow},1}=m[\left(h_{1}-h_{0}\right)-T_{0}\left(s_{1}-s_{0}\right)+{\mathrm{ke}}_{1}+{\mathrm{pe}}_{1}]$$

Exergy transfer associated with *shaft* and *electrical work* is the work itself, but exergy transfer associated with *moving boundary work* is the useful work carried out (the difference between work carried out and the atmospheric work):

(13) $$X_{W}=W_{b}-W_{\mathrm{surr}}\;\;(\mathrm{boundary}\;\mathrm{work})$$

(14) $$X_{W}=W\;\;(\mathrm{other}\;\mathrm{forms}\;\mathrm{of}\;\mathrm{work})$$

The phrases *work* and *useful work* are synonymous for steady-flow systems since they do not involve moving boundary work.

Exergy transfer associated with *heat transfer* at the system boundary at temperature *T_b_* is the amount of heat transfer *Q* multiplied by the Carnot efficiency:

(15) $$X_{Q}=\left(1-\frac{T_{0}}{T_{b}}\right)Q$$

The direction of exergy transfer with work is the same as the direction of work. This is also the case for heat transfer for systems whose temperature is above the environmental temperature. But the reverse is true for systems below the environment temperature since heat transfer to a system below the environment temperature decreases its exergy. So, it is not surprising that the equation above gives a *negative value* for exergy transfer *X_Q_* associated with heat input *Q* when *T_b_* < *T*_0_. Restoring the cold system to its original state requires the reversal of the heat gain process by removing heat in the amount of *Q* from the cold system, which can be accomplished using a Carnot refrigerator operating between the temperatures of *T_b_* and *T*_0_. The required work input of this *reversible refrigerator* is calculated as

(16) $$W_{\mathrm{rev},\mathrm{in}}=\frac{Q_{L}}{{COP}_{\mathrm{rev}}}=\frac{Q_{L}}{T_{L}/(T_{H}-T_{L})}=\frac{(T_{H}-T_{L})Q_{L}}{T_{L}}=\frac{(T_{0}-T_{b})Q_{L}}{T_{b}}=-\left(1-\frac{T_{0}}{T_{b}}\right)Q_{L}=-X_{Q\_L}$$

which is equivalent to the exergy transfer associated with heat transfer *Q_L_* to the cold medium, except in the reverse direction. Exergy transfer with heat transfer becomes zero when *T_b_* = *T*_0_. Therefore, exergy transfer associated with heat loss from an *extended system* (system + the immediate surroundings where gradients occur) is zero since the *boundary temperature* of an extended system is equal to the environmental temperature *T*_0_.

Considering that energy is *conserved*, entropy is *generated* (but never destroyed, *S*_gen_ ≥ 0), and exergy is *destroyed* (but never generated, *X*_destroyed_ ≥ 0), *energy, entropy, and exergy balances* for a general system undergoing any process can be written as follows:

*E*_in_ − *E*_out_ = Δ*E*_sys_ = (*E*_2_ − *E*_1_)_sys_ (17)

*S*_in_ − *S*_out_ + *S*_gen_ = Δ*S*_sys_ = (*S*_2_ − *S*_1_)_sys_ (18)

*X*_in_ − *X*_out_ − *X*_destroyed_ = Δ*X*_sys_ = (*X*_2_ − *X*_1_)_sys_ (19)

*Entropy generation S*_gen_ and *exergy destruction X*_destroyed_ are related to each other as follows:

*X*_destroyed_ =*T*_0_*S*_gen_ (20)

Both energy and exergy are transferred into and out of the system, in general by heat, work, and mass flow, while entropy is transferred by heat and mass (work is entropy free). Exergy destroyed *X*_destroyed_ depletes the exergy of the system (and thus it has the same sign as the exergy output *X*_out_), while entropy generated *S*_gen_ adds to the entropy of the system (and thus it has the same sign as the entropy input *S*_in_). *Energy, entropy,* and *exergy balances* can be expressed more explicitly as follows:

(*Q*_in_ − *Q*_out_) + (*W*_in_ − *W*_out_) = (*E*_2_ − *E*_1_)_sys_ (21)

(*S_Q_*_,in_ − *S_Q_*_,out_) + (*S*_mass,in_ − *S*_mass,out_) + *S*_gen_ = (*S*_2_ − *S*_1_)_sys_ (22)

(*X_Q_*_,in_ − *X_Q_*_,out_) + (*X_W_*_,in_ − *X_W_*_,out_) + (*X*_mass,in_ − *X*_mass,out_) − *X*_destroyed_ = (*X*_2_ − *X*_1_)_sys_ (23)

If the process between states 1 and 2 was *reversible*, there would be no exergy destruction (*X*_destroyed_ = 0) and the net useful work output (*W*_net,out_ = *X_W_*_,out_ − *X_W_*_,in_) in the exergy balance equation would be the reversible work output *W*_rev,out_ for that process. Then, the *reversible work output* for a process during which a system undergoes a process from state 1 to state 2 would become

*W*_rev,out_ = (*X_Q_*_,in_ − *X_Q_*_,out_) + (*X*_mass,in_ − *X*_mass,out_) + (*X*_1_ − *X*_2_)_sys_ (24)

Reversible work output *W*_rev,out_ for a process represents the maximum useful work that can be carried out during that process. That is, for a system that may involve heat transfer with many heat reservoirs and mass flows entering and exiting, the maximum useful work that can be carried out is the *sum* of (1) the net exergy transfer into the system by heat transfer, (2) the net exergy transfer into the system by mass flow, and (3) the decrease in the exergy of the system. The *actual* useful work output for the process will be lower. The difference between the reversible work output and the actual useful work output is the exergy destroyed, *X*_destroyed_ = *W*_rev,out_ − *W*_act,out_, also called *lost work*.

A negative value for *W*_rev,out_ indicates *reversible work input W*_rev,in_, which is the minimum work input required for the process to take place. In this case, exergy destroyed is the difference between the actual useful work input and the reversible work input, *X*_destroyed_ = *W*_act,in_ − *W*_rev,in_.

The general *exergy balance* and *reversible work* relations can also be written in the *rate form* as follows:

(25) $$({\dot{X}}_{Q,\mathrm{in}}-{\dot{X}}_{Q,\mathrm{out}})+({\dot{X}}_{W,in}{\dot{X}}_{W,out})+({\dot{X}}_{\mathrm{mass},\mathrm{in}}-{\dot{X}}_{\mathrm{mass},\mathrm{out}})-{\dot{X}}_{\mathrm{destroyed}}=d{\dot{X}}_{\mathrm{sys}}/dt$$

(26) $${\dot{W}}_{\mathrm{rev},\mathrm{out}}=\left({\dot{X}}_{Q,in}-{\dot{X}}_{Q,out}\right)+\left({\dot{X}}_{\mathrm{mass},\mathrm{in}}-{\dot{X}}_{\mathrm{mass},\mathrm{out}}\right)-d{\dot{X}}_{\mathrm{sys}}/dt$$

where the overdot denotes *time rate*.

## 3. Chemical Exergy

*Mixing* and *separation* processes are commonly used in practice. Mixing of gases or liquids occurs spontaneously and involves entropy generation and thus exergy destruction since *X*_destroyed_ = *T*_0_*S*_gen_. This means, if done reversibly, useful work can be obtained during *mixing*, and destroyed exergy can be recovered as work. It also means that the reverse process of *separation* requires work input. In the reversible case, the required work input for a separation process is equal to the work that can be produced during the reverse process of mixing.

Gas mixtures or liquid solutions can be treated as *ideal mixtures* or *ideal solutions* if the influence of dissimilar molecules on each other can be neglected. Below, we first consider the chemical exergy associated with concentration change in the absence of any chemical reactions, followed by the chemical exergy associated with chemical reactions.

The *specific Gibbs function* (or *Gibbs free energy*) *g* is defined as the combination property *g* = *h − Ts.* The total Gibbs function *G* of a mixture of *k* components is a function of two independent properties and the composition of the mixture, and is expressed as *G* = *G*(*T*, *P*, *N*_1_, *N*_2_, … *N_k_*). Its total differential can be expressed as [1]

(27) $$dG=VdP-SdT+\Sigma \mu_{i}dN_{i}$$

and dividing by the total number of moles of the mixture *N_m_* = *N*_1_, *N*_2_, … *N_k_* gives

(28) $$d\bar{g}=\bar{v}dP-\bar{s}dT+\Sigma \mu_{i}dy_{i},$$

where ***V*** is total volume, *y_i_* = *N_i_*/*N_m_* is the *mole fraction* of component *i*, overbar denotes properties per unit mole, and

(29) $${\bar{\mu }}_{i}={\left(\partial G/\partial N_{i}\right)}_{P,T,N_{j}}={\bar{g}}_{i}={\bar{h}}_{i}-T{\bar{s}}_{i}$$

is the *chemical potential* of component *i,* which is the *differential change in the total Gibbs function* of the mixture in a specified phase per differential change of component *i* in the same phase of mixture *P* and *T* while the mole numbers of all other components *N_j_* are held constant. For a single-component system, the last term in Equation (26) with the summation sign drops out since the composition is fixed. Thus, in a specified phase, the chemical potential of a pure substance is equivalent to its Gibbs function since $G=N\bar{g}=\;N\bar{\mu }$, where

(30) $${\bar{\mu }}_{\mathrm{pure}\;\mathrm{substance}}={\left(\partial G/\partial N\right)}_{P,T}=\bar{g}=\bar{h}-T\bar{s}$$

Therefore, the difference between the chemical potential and the Gibbs function is due to the effect of *dissimilar molecules* in a mixture on each other.

When the effect of dissimilar molecules on each other in a mixture is negligible, the mixture is said to be an *ideal mixture* or *ideal solution* and the chemical potential of a component in such a mixture equals the Gibbs function of the pure component. Many liquid solutions encountered in practice, especially dilute ones, satisfy this condition closely and can be considered ideal solutions with negligible error.

When the kinetic and potential energies are negligible, the *chemical exergy* of a flowing pure substance on a unit mass basis can be determined from the flow exergy relation in Equation (7), *x*_flow_ = (*h − h*_0_) − *T*_0_(*s − s*_0_), by rearranging it as *x*_flow_ = (*h − T*_0_*s*) − *T*_0_(*h*_0_ − *T*_0_*s*_0_). When the temperature *T* of the pure substance equals the reference environmental temperature *T*_0_, *chemical exergy* becomes

*x*_ch,pure,@*T*=*T_*0_ = *x*_flow,pure_ = [(*h − T*_0_*s*) − (*h*_0_ − *T*_0_*s*_0_)]_@*T_*0_ = [*g* − *g*_0_]_@*T_*0_ = [*μ* − *μ*_0_]_@*T_*0_ (31)

Therefore, the *chemical exergy* of a flowing substance at the environmental temperature of *T*_0_ is equal to the difference between the *Gibbs function* or *chemical potential* of the system at the given pressure *P* and temperature *T*_0_ and in the dead state of *P*_0_ and *T*_0_. Note that in the dead state, the mole fraction of the substance is equal to its mole fraction in the reference environment. For a *component i* in an ideal mixture or ideal solution, *chemical exergy* is expressed as

*x*_ch*,i*,*T*=*T_*0_ = *x*_flow,*i*_ = [(*h_i_ − T*_0_*s_i_*) − (*h_i_*_,0_ − *T*_0_*s_i_*_,0_)]*_T__*_0_ = [*g_i_* − *g_i_*_,0_]_@*T_*0_ = [*μ_i_* − *μ_i_*_,0_]_@*T_*0_ (32)

The *standard environment* in a gas phase is typically represented by the atmospheric air at *T*_0_ = 25 °C and *P*_0_ = 1 atm with fixed mole fractions of *y_i_*_,env_ (and thus, from Dalton’s law, partial pressures of *P_i_*_,env_ = *y_i_*_,env_*P*_0_) for the components N_2_, O_2_, CO_2_, H_2_O (*g*), O_3_, NO*_x_*, etc. Note that ∑*y_i_*_,env_ = 1 and ∑*P_i_*_,env_ = *P*_0_. Therefore, a component in a gas mixture at temperature *T*_0_ and mole fraction of *y_i_* (and thus partial pressure of *P_i_* = *y_i_P*_0_) will reach chemical equilibrium with the environment when its mole fraction *y_i_* becomes equal to the corresponding environmental value of *y_i_*_,env_ (and thus its partial pressure *P_i_* becomes equal to the environmental value of *P_i_*_,env_).

The *chemical exergy x*_ch*,i*_ of a component *i* in a gas mixture in a restricted dead state of *T*_0_ and *P*_0_ is the maximum work that can be carried out as the gas *i* expands steadily from its partial pressure *P_i_* to the corresponding partial pressure in the environment *P_i_*_,env_ during a reversible isothermal process at the constant temperature of *T*_0_. For an ideal gas undergoing a steady-flow isothermal process from a state of *T*_0_ and *P_i_* to a state of *T*_0_ and *P_i_*_,env_ the *chemical exergy x*_ch*,i*_ of a component *i* in a gas mixture is determined to be

(33) $${\bar{x}}_{\mathrm{ch},i}=\left({\bar{h}}_{i}-{\bar{h}}_{i,0}\right)-T_{0}\left({\bar{s}}_{i}-{\bar{s}}_{i,0}\right)=0-T_{0}\left(c_{p,i}ln\frac{T_{i}}{T_{i,0}}-R_{u}ln\frac{P_{i}}{P_{i,0}}\right)=T_{0}R_{u}ln\frac{y_{i}}{y_{i,\mathrm{env}}}$$

since the temperature is constant and the enthalpy of an ideal gas remains constant at a constant temperature. Here, *R_u_* = 8.314 kJ/kmol·K is the universal gas constant.

Alternately, noting that *v* = *RT/P* for an ideal gas and the isothermal steady-flow reversible work output is *W*_rev,out_ = −ʃ*vdP* = −*RT*ln(*P*_2_/*P*_1_), the *chemical exergy* of component *i* in an ideal gas mixture per unit mole becomes

(34) $${\bar{x}}_{\mathrm{ch},i}={\bar{w}}_{\mathrm{rev},\mathrm{out}}=-R_{u}T_{0}ln\frac{P_{i,\mathrm{env}}}{P_{i}}=-R_{u}T_{0}ln\frac{y_{i,\mathrm{env}}P_{0}}{y_{i}P_{0}}=-R_{u}T_{0}ln\frac{y_{i,\mathrm{env}}}{y_{i}}$$

which is identical to the relation obtained earlier. The *standard chemical exergy* ${\bar{x}}_{\mathrm{ch}}^{0}$ of a pure substance at *T*_0_ and *P*_0_ can be obtained similarly by setting *y_i_* = 1 in the relation above,

(35) $${\bar{x}}_{\mathrm{ch}.\mathrm{pure}@T0,\;P0}={\bar{x}}_{\mathrm{ch}}^{0}=-R_{u}T_{0}lny_{i,\mathrm{env}}$$

where the superscript 0 denotes the standard reference conditions of *T*_0_ and *P*_0_. The chemical exergy of the gas mixture per unit mole of mixture in the restricted dead state of *T*_0_ and *P*_0_ is the sum of the chemical exergies $N_{i}{\bar{x}}_{\mathrm{ch},i}$ of the components divided by the total number of moles of the mixture. Noting that *y_i_* = *N_i_*/*N_m_*, we obtain the following:

(36) $${\bar{x}}_{\mathrm{ch},\mathrm{mixture}}=\sum \frac{N_{i}}{N_{m}}{\bar{x}}_{\mathrm{ch},i}=\sum y_{i}{\bar{x}}_{\mathrm{ch},i}=-R_{u}T_{0}\sum y_{i}ln\frac{y_{i,\mathrm{env}}}{y_{i}}$$

Also noting that ln(*y_i,_*_env_/*y_i_*) = ln(*y_i,_*_env_) − ln(*y_i_*) and ${\bar{x}}_{\mathrm{ch},i}^{0}=-R_{u}T_{0}lny_{i,\mathrm{env}}$, the equation above can be expressed in terms of *pure substance standard chemical exergies of components* instead of mole fractions in the environment as follows [32,33,34]:

(37) $${\bar{x}}_{\mathrm{ch}.\mathrm{mixture}}=\sum y_{i}{\bar{x}}_{\mathrm{ch},i}^{0}+R_{u}T_{0}\sum y_{i}lny_{i}$$

This equation has the advantage that it can be used for gas mixtures that contain gases that are not included in the reference environment such as a gaseous fuel or combustion products, and the standard chemical exergy values of pure substances are readily available in the tables. For example, taking the mole fraction of the CO_2_ in the environment to be 0.0003, the *standard chemical exergy* of CO_2_ is determined to be

$${\bar{x}}_{\mathrm{CO}2}^{0}=-R_{u}T_{0}lny_{\mathrm{CO}2,\mathrm{env}}=-\left(8.314\mathrm{kJ}/\mathrm{kmol}\cdot K\right)\left(298K\right)\ln\left(0.0003\right)=20,100\mathrm{kJ}/\mathrm{kmol}.$$

That is, when 1 kmol of CO_2_ at *T*_0_ and *P*_0_ is allowed to mix with the standard atmosphere with a CO_2_ mole fraction of 0.03 percent, there is the potential to produce 20,100 kJ of work. As another example, the total chemical exergy of combustion products can be expressed as

(38) $$X_{\mathrm{ch}.\mathrm{products}}=N_{p}{\bar{x}}_{\mathrm{ch}.\mathrm{products}}=\sum N_{p,i}{\bar{x}}_{\mathrm{ch},i}=\sum N_{p,i}{\bar{x}}_{\mathrm{ch},i}^{0}+R_{u}T_{0}\sum N_{p.i}lny_{i}$$

where *N_p,i_* is the mole number of product *i*, *N_p_* = ∑*N_i_* is the total number of moles of combustion products, and *y_i_* = *N_p,i_*/*N_p_* is the mole fraction of product *i*. This represents the work that can be produced if the combustion products are allowed to mix with the environment in a reversible manner until each product reaches the concentration level in the environment and thus achieves chemical equilibrium with the environment.

Likewise, the total chemical exergy of reactants can be expressed as

(39) $$X_{\mathrm{ch}.\mathrm{reactants}}=N_{r}{\bar{x}}_{\mathrm{ch}.\mathrm{reactants}}=\sum N_{r,i}{\bar{x}}_{\mathrm{ch},i}=\sum N_{r,i}{\bar{x}}_{\mathrm{ch},i}^{0}+R_{u}T_{0}\sum N_{r,i}lny_{i}$$

where *N_r,i_* is the mole number of reactant *i*, *N_r_* = ∑*N_r_* is the total number of moles of reactants, and *y_i_* = *N_r,i_*/*N_r_* is the mole fraction of reactant *i*.

The exergy relations above are developed under the assumption that the gas mixture behaves as an ideal gas, but they can be used for real gas mixtures that closely approximate ideal gases. They can also be used for liquid solutions that can be approximated as ideal solutions with negligible error.

Exergy is a measure of work potential, and thus the chemical exergy relations given above are also relations for reversible work. Therefore, assuming ideal mixtures or ideal solutions, the *maximum possible work* that can be obtained from a mixture at *T*_0_ and *P*_0_ as it reaches chemical equilibrium with the environment is

(40) $$W_{\mathrm{rev},\mathrm{out},\mathrm{mixing}}=N_{m}{\bar{x}}_{\mathrm{ch}.\mathrm{mixture}}=-N_{m}R_{u}T_{0}\sum y_{i}ln\frac{y_{i,\mathrm{env}}}{y_{i}}=N_{m}\left(\sum y_{i}{\bar{x}}_{\mathrm{ch},i}^{0}+R_{u}T_{0}\sum y_{i}lny_{i}\right)$$

or, noting that *N_i_* = *y_i_N_m_*,

(41) $$W_{\mathrm{rev},\mathrm{out},\mathrm{mixing}}=\sum N_{i}{\bar{x}}_{\mathrm{ch},i}^{0}+R_{u}T_{0}\sum N_{i}lny_{i}$$

When a reversible process is reversed, the directions of interactions are also reversed, but magnitudes remain the same. Therefore, the relation above also gives the minimum work input required to separate the components from the environment and form the original mixture with mole fractions *y_i_*. That is, $W_{\mathrm{rev},\mathrm{in},\mathrm{separation}}=W_{\mathrm{rev},\mathrm{out},\mathrm{mixing}}$.

### Chemical Exergy Associated with Chemical Reactions

The chemical exergy relations given above are limited to systems that involve concentration changes but no chemical reactions. Also, the systems are at temperature *T*_0_ and pressure *P*_0_ of the environment. Those relations are sufficient to determine the chemical exergy of mixtures with no regard to the exergy associated with the formation of the components *x_i,f_* since they cancel out when there are no chemical reactions. When *T* = *T*_0_, the exergy of a substance becomes equal to its Gibbs function, as discussed above. Therefore, the exergy associated with the formation of a substance is simply the *Gibbs function of formation* of that substance in the standard reference state, ${\bar{x}}_{i,\mathrm{formation}}={\bar{g}}_{f,i}^{0}$. It is the *reversible work* produced or required as the substance is formed from its stable elements at the standard reference state of *T*_0_ and *P*_0_ (the Gibbs function of formation of stable elements such as O_2_ and N_2_ in the standard reference state are zero). Then, it follows that in the presence of chemical reactions, the chemical exergy of a component *i* is obtained by adding the exergy of formation to the chemical exergy relations given above:

(42) $${\bar{x}}_{\mathrm{ch}.\mathrm{mixture}}=\sum y_{i}{\bar{g}}_{f,i}^{0}+\sum y_{i}{\bar{x}}_{\mathrm{ch},i}=\sum y_{i}{\bar{g}}_{f,i}^{0}+\sum y_{i}{\bar{x}}_{\mathrm{ch},i}^{0}+R_{u}T_{0}\sum y_{i}lny_{i}$$

This is similar to adding the standard *enthalpy of formation* ${\bar{h}}_{f}^{0}$ terms to the enthalpy expressions in energy balances when the process involves chemical reactions and using ${\bar{h}}_{f}^{0}+\bar{h}-{\bar{h}}_{0}$ instead of $\bar{h}-{\bar{h}}_{0}$. Values of standard Gibbs functions of formation of a substance can be obtained from property tables just like the enthalpy of formation values. Then, the chemical exergy of reactants and products of a chemical reaction can be expressed as follows:

(43) $$X_{\mathrm{ch}.\mathrm{reactants}}=\sum N_{r,i}{\bar{g}}_{f,i}^{0}+\sum N_{r,i}{\bar{x}}_{\mathrm{ch},i}^{0}+R_{u}T_{0}\sum N_{r,i}lny_{i}$$

(44) $$X_{\mathrm{ch}.\mathrm{products}}=\sum N_{p.i}{\bar{g}}_{f,i}^{0}+\sum N_{p.i}{\bar{x}}_{\mathrm{ch},i}^{0}+R_{u}T_{0}\sum N_{p.i}lny_{i}$$

If the reactants enter at *T*_1_ and *P*_1_ instead of *T*_0_ and *P*_0_ and the kinetic and potential energies are considered, exergy change ${\bar{X}}_{1}-{\bar{X}}_{0}=\sum N_{i}{\left[\left({\bar{h}}_{1}-{\bar{h}}_{0}\right)-T_{0}\left({\bar{s}}_{1}-{\bar{s}}_{0}\right)+{\overset{-}{\mathrm{ke}}}_{1}+{\overset{-}{\mathrm{pe}}}_{1}\right]}_{i}$ associated with this change of state should be added to the exergy of the reactants. The same can be said for the products.

The difference between the two gives *reversible work output* associated with a stead-flow chemical reaction, which is the maximum amount of work that can be carried out during that reaction when all reactants enter and products leave at the standard reference temperature *T*_0_ and pressure *P*_0_ with negligible kinetic and potential energies:

(45) $$\begin{array}{l} {W_{\mathrm{rev},\mathrm{out},\mathrm{reaction}}=X}_{\mathrm{ch}.\mathrm{reactants}}-X_{\mathrm{ch}.\mathrm{products}}\\ =\left(\sum N_{r,i}{\bar{g}}_{f,i}^{0}+\sum N_{r,i}{\bar{x}}_{\mathrm{ch},i}^{0}+R_{u}T_{0}\sum N_{r,i}lny_{i}\right)-\left(\sum N_{p,i}{\bar{g}}_{f,i}^{0}+\sum N_{p,i}{\bar{x}}_{\mathrm{ch},i}^{0}+R_{u}T_{0}\sum N_{p.i}lny_{i}\right) \end{array}$$

It can also be expressed as

(46) $$W_{\mathrm{rev},\mathrm{out},\mathrm{reaction}}={∆G}_{f}^{0}+\left(\sum N_{r,i}{\bar{x}}_{\mathrm{ch},i}^{0}+R_{u}T_{0}\sum N_{r,i}lny_{i}\right)-\left(\sum N_{p.i}{\bar{x}}_{\mathrm{ch},i}^{0}+R_{u}T_{0}\sum N_{p.i}lny_{i}\right)$$

where:

$${∆G}_{f}^{0}=\sum N_{r,i}{\bar{g}}_{f,i}^{0}-\sum N_{p,i}{\bar{g}}_{f,i}^{0}$$

A negative value for *W*_rev,out, reaction_ indicates the required work input.

## 4. Definition of Exergy Efficiency

*Exergy efficiency* or *second-law efficiency* is a measure of the actual performance of an energy system relative to the thermodynamically best possible performance, which occurs under reversible conditions characterized by zero entropy generation or zero exergy destruction. Then, it follows that the exergy efficiency of all reversible processes or devices is 100 percent.

A general form of exergy or second-law efficiency is commonly expressed as the ratio of the exergy output *X*_out_ to the exergy input *X*_in_ [35]:

(47) $$\eta_{\mathrm{ex}}=\frac{\mathrm{Exergy}\;\mathrm{output}}{\mathrm{Exergy}\;\mathrm{input}}=\frac{X_{\mathrm{out}}}{X_{\mathrm{in}}}=1-\frac{X_{\mathrm{destroyed}}+X_{\mathrm{loss}}}{X_{\mathrm{in}}}$$

This definition compares the exergy that leaves the system as the *desirable output* or *valuable product* to the exergy supplied to the system as the *required input* or *invested commodity* in the currency of exergy. The difference between the exergy input and output is the exergy destroyed and exergy lost, *X*_in_ − *X*_out_ = *X*_destroyed_ + *X*_loss_. Some definitions [36] do not include the *X*_loss_ term in the second part of the exergy efficiency expression above.

There is often uncertainty as to what constitutes *exergy output* and *exergy loss* and how they can be differentiated. The answer depends on the intended purpose of the system being analyzed and its role in the operation. The exergy output associated with heat loss to the environment is clearly exergy loss, but the exergy output that accompanies heat transfer to a preheater or cogeneration system is not. The kinetic, chemical, and thermal exergies associated with gases emitted to the environment are also exergy loss. Double-counting should be avoided, and exergy book-keeping should be observed. If an outgoing exergy term is labeled as exergy loss, it can no longer be included in the exergy output. Also, as the phrases *exergy input* and *exergy output* imply, the exergy efficiency relation above is intended for steady-flow systems since the exergy content of such systems remains constant. Therefore, if it is used for closed and unsteady-flow systems, the exergy change of the system should be accounted for as exergy input or output, as appropriate.

Both *exergy destruction* and *exergy loss* represent a waste of exergy, unless the lost exergy is supplied as input to another system. Wasted exergy quantity is regarded either as exergy loss or exergy destruction, depending on the choice of system boundary and thus the specification of the system. Exergy associated with *heat loss* from a device will be counted as *exergy loss* if the physical system is taken as the system. But it will be counted as exergy destruction if the *extended system* is taken as the system since the exergy loss that accompanies heat loss will be destroyed in the immediate surroundings in that case and will become part of the exergy destroyed within the system. Likewise, the exergy content of *exhaust gases* (thermal, chemical, and kinetic exergy) will be treated as *exergy loss* if the physical device is taken as the system, but as *exergy destruction* if the extended system is taken as the system since the temperature, pressure, velocity, and chemical composition of exhaust gases will be equal to those of the environment at the boundary of the extended system. Considering that exergy loss is eventually destroyed, it makes practical sense to work with the extended system and treat both quantities as exergy destruction to avoid this complication.

Once *X*_in_ and *X*_out_ are calculated, the equation above will always give an exergy efficiency value of less than 100 percent for all processes that involve irreversibilities. A system with *high exergy efficiency* has *low exergy destruction* and thus it approximates reversible operation more closely. Also, a decision has to be made as to what is to be counted as the exergy input and as exergy output by considering the intended outcome of the system and the required inputs. Different decisions may lead to different exergy efficiency expressions, and it is important to come up with the definition that is most meaningful and provides the best insight.

Exergy efficiency is also defined as the ratio of the *product* to the *fuel* [4] as follows:

(48) $$\eta_{\mathrm{ex}}=\frac{\mathrm{Product}}{\mathrm{Fuel}}=\frac{X_{\mathrm{product}}}{X_{\mathrm{fuel}}}=1-\frac{X_{\mathrm{destroyed}}+X_{\mathrm{loss}}}{X_{\mathrm{fuel}}}$$

Here, the *product* represents the desired output in terms of *exergy produced* by the system, and thus the identified product should reflect the purpose of purchasing the system. The *fuel* represents the *exergy resources expended* to generate the product and is not limited to actual fuel. Therefore, exergy efficiency is the percentage of the fuel exergy supplied that is found in the product exergy. Exergy destruction and exergy loss represent the portion of the fuel exergy wasted in the system. The two exergy definitions above are practically identical since there exists sufficient flexibility on deciding what constitutes output/input and product/fuel. Again, this exergy efficiency definition is also intended for steady-flow systems, and thus the exergy change of the system should be accounted for properly as fuel or product if it is used for closed and unsteady-flow systems. Of the two definitions, we will refer to the exergy input–output in the analysis since the terms *exergy input* and *exergy output* are more descriptive than the terms *fuel* and *product*.

An alternative form of general exergy or second-law efficiency is expressed as the ratio of the *exergy expended* (or consumed) *X*_expended_ to the *exergy recovered X*_recovered_ [1],

(49) $$\eta_{\mathrm{ex}}=\frac{\mathrm{Exergy}\;\mathrm{recovered}}{\mathrm{Exergy}\;\mathrm{expended}}=\frac{X_{\mathrm{recovered}}}{X_{\mathrm{expended}}}=1-\frac{X_{\mathrm{destroyed}}}{X_{\mathrm{expended}}}$$

where exergy destroyed is the difference between expended and recovered exergy, and $X_{\mathrm{destroyed}}=X_{\mathrm{expended}}-X_{\mathrm{recovered}}$. This definition differs from those above in that it does not involve the term *exergy loss*.

When the physical device is taken as the system, *recovered exergy* includes the exergy that is eventually lost (associated with heat loss and mass crossing the system boundary at the outlets), and thus the deviation of exergy efficiency from 100% serves as a measure of *exergy destruction* within the system boundaries. Also, the increase in the exergy of the system is included in *recovered exergy,* while the decrease in the exergy of the system is included in *expended exergy*. Therefore, the phrase *expended–recovered exergy* appears to be more appropriate and general compared to the phrases *exergy input–output* and *fuel–product exergy* since the former establishes a more inclusive basis for the evaluation of the exergy efficiency of closed and unsteady-flow systems. From a practical perspective, all approaches allow engineers to decide what is to be included as expended and recovered exergy, exergy input and output, and fuel and product exergy by considering what the device or system is intended for and the particulars of the operation.

In the exergy expended–recovered approach, the exergy resources utilized such as heat transfer, mechanical or electrical work supply, incoming fluid streams, or exergy content of the system are identified first. Work consumed by the system is always part of exergy expended. When part of the exergy content of the system is consumed during a process, it is also counted as exergy expended. For a fluid stream, exergy expended is the decrease in the exergy between the inlet and exit states. When a fluid stream leaves the system in the dead state and thus has zero exit exergy, the exergy expended associated with the stream is the inlet exergy of the stream. This is also the case for heat transferred to the system when all heat loss occurs at the environmental temperature.

When determining exergy efficiency with the exergy expended–recovered approach, first we need to determine how much exergy is expended or consumed during the process. In a reversible operation, we recover the entire exergy expended during the process and the exergy destroyed is zero. The exergy efficiency of this process is 100 percent. When we recover none of the exergy expended, exergy efficiency becomes zero. Note that exergy can be supplied or recovered in various forms such as heat, work, mass of system, and fluid streams. *Expended exergy* is the *net exergy supplied* to the system via heat, work, and mass flow to accomplish the intended process, plus the decrease in the exergy of the system for closed and unsteady-flow systems. *Recovered exergy* is the difference between the expended exergy and the exergy destroyed during the process.

The system analyzed should be well defined to identify exergy interactions between the system and its surroundings accurately. For a closed-cycle heat engine, exergy expended is the exergy of the heat transferred to the engine, while recovered exergy is the net work produced by the engine plus the exergy of the heat rejected. If heat is rejected at the environmental temperature, the exergy of the heat rejected is zero. In this case, the exergy expended becomes equal to the exergy input by heat, while the exergy recovered becomes the net work output.

For a refrigerator or heat pump, *expended exergy* is usually the work consumed, which is identical to the exergy input. *Recovered exergy* is the exergy of the heat transferred to the high-temperature medium for a heat pump and the exergy of the heat transferred from the low-temperature medium for a refrigerator, which are identical to the exergy output.

In exergy analysis, we also use the reversible work approach as appropriate, especially for steady-flow devices that involve work-producing devices such as turbines and work-consuming devices such as compressors, pups, and fans:

(50) $$\eta_{\mathrm{ex},\;W\_\mathrm{out}}=\frac{W_{\mathrm{act},\;\mathrm{out}}}{W_{\mathrm{rev},\;\mathrm{out}}}\mathrm{and}\;\eta_{\mathrm{ex},\;W\_\mathrm{in}}=\frac{W_{\mathrm{rev},\;\mathrm{in}}}{W_{\mathrm{act},\;\mathrm{in}}}$$

Here, *W*_act_ is the actual work and *W*_rev_ is the reversible work.

For *extended systems*, all approaches usually give the same result for exergy efficiency. But this is not always the case when the *physical system* is taken as the system. Below, we determine the exergy efficiency of closed systems and unsteady-flow systems using the appropriate general definition given above. When deciding what constitutes expended exergy or exergy input and what constitutes recovered exergy or exergy output, it is important to keep in mind the purpose of the system and the operation. It makes thermodynamic sense to identify the sum of *all exergy inputs* as the exergy input or expended exergy and the sum of all exergy outputs as the exergy output or recovered exergy. But this may not be in line with the purpose of purchasing that system and the economic aspects. In the end, the calculated exergy efficiency should make the most sense and provide the best insight when judging the performance of the system.

As will be seen in the sections that follow, exergy efficiency based on the exergy expended–recovered approach is rather intuitive and can be applied to practically all processes including those associated with rigid tanks, piston–cylinder devices, and unsteady-flow devices. The exergy efficiency relations developed below are demonstrated with applications. Calculations are performed using EES software (Version 10) with built-in thermodynamic properties [37].

## 5. Novelty and Methodology

This paper represents an original contribution to thermodynamics and fills an important gap in the literature on the definition of exergy efficiency and its application to energy systems while clearing some common misconceptions. A literature review reveals that exergy efficiency relations for specific closed or unsteady-flow systems undergoing specified processes exist, but exergy efficiency relations for general closed and unsteady-flow systems do not exist. This void is remedied here by systematically developing the forementioned relations from the basics.

The root of the problems associated with exergy efficiency relations appears to be the relation ${\eta_{\mathrm{ex}}=X}_{\mathrm{out}}/X_{\mathrm{in}}$ or ${\eta_{\mathrm{ex}}=X}_{\mathrm{product}}/X_{\mathrm{fuel}}$ as the general definition of exergy efficiency and applying it to all systems. This fundamental relation works fine for systems in steady operation, but not so for closed systems and unsteady-flow devices without broad interpretations. The alternative general definition ${\eta_{\mathrm{ex}}=X}_{\mathrm{recovered}}/X_{\mathrm{expended}}$ works best for the latter cases. There is also uncertainty as to what forms of exergy constitute exergy input, exergy output, exergy expended, and exergy recovered. To obtain the most useful and meaningful results, these terms should be identified properly by considering the particulars of the system analyzed.

Most differences in exergy efficiency definitions can be traced to treating exergy transfer associated with heat loss and mass expelled to the environment as *exergy loss* or *exergy destroyed*, as indicated before. This confusion can be avoided by invoking and utilizing the concept of the *extended system,* which is the system plus its immediate surroundings where temperature and concentration gradients between the system and environment exist. The extended system includes the temperature and concentration gradient zones, and the boundary temperature, pressure, and concentration values are those of the environment. For example, when a rigid tank with a heat loss of *Q*_loss_ is taken as the system, the outer surface of the tank constitutes the system boundary, and the exergy transfer associated with heat loss (*X_Q_*__loss_) is determined from *X_Q_*__loss_ = (1 − *T*_0_/*T_b_*)*Q*_loss_, where *T_b_* is the average boundary temperature. The entire *X_Q_*__loss_ is destroyed within the immediate surroundings. Exergy destruction associated with heat loss within the immediate surroundings can also be determined from $X_{\mathrm{destroyed}}=T_{0}S_{\mathrm{gen}}$, where entropy generation is calculated from the entropy balance on the immediate surroundings, with $S_{\mathrm{gen}}=Q_{loss}/T_{0}-Q_{loss}/T_{b}$.

The term *X_Q_*__loss_ is part of the exergy leaving the tank when the physical tank is taken as the system. Unlike other exergy output terms, it is properly called *exergy loss* since exergy associated with heat loss is unlikely to be recovered and utilized for a useful purpose elsewhere. Consequently, exergy loss is usually destined to be exergy destroyed, but the destruction takes place outside the system boundaries. To properly account for the exergy destruction that occurs in the immediate surroundings, it should be made clear whether the immediate surroundings of the system are included as part of the system or as part of the surroundings.

The treatment of exergy loss *X*_loss_ is a source of confusion since some consider it part of the exergy output while others group it with the exergy destruction term in the evaluation of exergy efficiency. The analysis of the extended system bypasses all this confusion by doing away with the concept of *exergy loss* and thus treating the potentiality of the eventual destruction of exergy loss as actuality. In the case of the *extended system*, the exergy loss is zero since the exergy contents of thermal energy and purged substances in the environmental conditions are zero. If we want to determine the fraction of exergy destruction due to exergy loss, it can be determined easily by dividing *exergy loss* by the *exergy expended* (or its equivalent exergy input), *X*_loss_/*X*_expended_.

This approach simplifies the analysis and eliminates the need to distinguish between exergy loss and exergy destruction by including the exergy loss associated with heat loss and purged substances in exergy destruction. The exergy efficiency evaluated this way is also more realistic since it properly accounts for all exergy destruction associated with the process, including the exergy destroyed within the immediate surroundings which is unavoidable. Therefore, the exergy efficiency of the *extended system* is equivalent to the exergy efficiency of the *process*. The extended system approach can also be used for systems whose outer surfaces are covered with thermoelectric generators by simply including the work produced by them as part of the exergy output. For adiabatic systems, the choice of the actual system or extended system is irrelevant since the immediate surroundings shrink to zero in this case.

When the analyzed system involves substances purged to the environment as part of the exiting mass as well as heat loss, the immediate surroundings include the concentration gradients as well as temperature gradients between the system and the environment. The purged matter mixes with the matter in the immediate surroundings such that the composition at the boundary of the extended system becomes the composition of the environment. That is, each component of the purged mass undergoes a change from the state of the purging site to the state of the environment (the dead state), including its concentration. This way, the exergy of each component, including the chemical exergy, is zero, and *X*_purged mass_ = 0 at the extended system boundary. When the physical system is taken as the system, the exergy transfer associated with purged mass constitutes exergy loss, *X*_loss, purged mass_. This exergy is completely destroyed in the immediate surroundings, and the exergy destruction associated with the purged mass within the immediate surroundings is its exergy content, *X*_destroyed, purged mass_ = *X*_loss, purged mass_$\;=T_{0}S_{\mathrm{gen}}$, where entropy generation is calculated from the entropy balance of the immediate surroundings. The quantity *X*_purged mass_ or *X*_loss, purged mass_ represents the work potential of the purged mass at the boundary of the physical system, which is eventually destroyed within the immediate surroundings.

In the analysis below, we develop exergy efficiency relations for the physical system by considering exergy transfer by heat loss *X_Q_*__loss_ as part of the exergy recovered. The exergy efficiency relations for the corresponding extended system or process can be obtained by setting this term equal to zero, *X_Q_*__loss_ = 0. Exergy transfer associated with the purged mass does not appear in formulations explicitly since it is treated as one of the fluid streams exiting the system, *X*_purged mass_ = *m*_purged mass_ *x*_purged mass_, where *x*_purged mass_ includes the chemical exergy. For an extended system, it is zero, *X*_purged mass_ = 0. For simplicity, we treat the exergy loss associated with heat loss or mass purged to the environment as part of the exergy recovered in the analysis and avoid the use of the term *exergy loss* altogether since it is already accounted for. Again, its share of exergy destruction can be determined easily by dividing it by the exergy expended or exergy input.

To show the convenience of the use of an extended system in the exergy or second-law analysis, consider a *compressed-air storage system* with air being discharged through a turbine, producing power. There is no apparent ‘exergy input’ to the system, and thus the relation ${\eta_{\mathrm{ex}}=X}_{\mathrm{out}}/X_{\mathrm{in}}$ does not seem appropriate for use in this case unless exergy input is interpreted to include the decrease in the exergy of the system. Instead, we use the relation ${\eta_{\mathrm{ex}}=X}_{\mathrm{recovered}}/X_{\mathrm{expended}}$. If we analyze the *extended system* that includes the air storage tank, the turbine, and their immediate surroundings, the decrease in the exergy of the compressed air becomes *X*_expended_, and the work output of the turbine becomes *X*_recovered_. Their ratio gives the exergy efficiency of this process, which represents the fraction of the consumed exergy that is converted to mechanical work. In practice, this is what we care about the most—how close we are to an exergy efficiency of 100%. The calculated exergy efficiency value includes the effects of any heat loss from the tank and the turbine and thus the exergy destroyed within their immediate surroundings. Analyzing the extended system simplifies the analysis considerably since it alleviates the need to calculate the exergy transfer associated with heat loss and the purged air or to make the assumption of adiabatic operation.

## 6. Exergy Efficiency of Closed Systems

**Figure 1:**
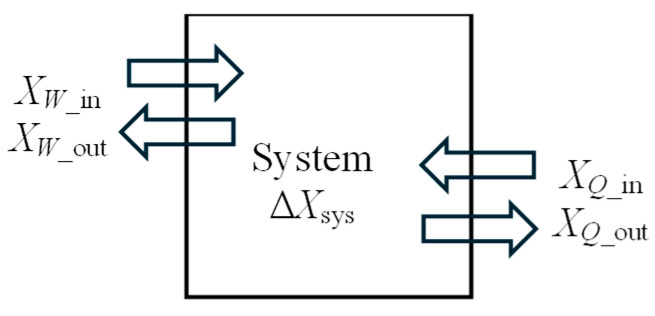
Exergy interactions associated with closed system.

In this section, we develop exergy efficiency relations for closed systems such as rigid tanks and piston–cylinder devices of fixed mass *m* using the approaches given above. A closed system may involve exergy input by heat and work, but not by mass flow (Figure 1). The exergy content of the system may increase or decrease during the process, depending on the direction of the net exergy transfer by heat and work. Also, the exergy content of the system may serve as an exergy resource by producing work or supplying heat.

For an adiabatic piston–cylinder device that is expanding and producing work, the exergy of the system itself is used as the exergy resource, and the decrease in the exergy of the system constitutes exergy expended. The useful work produced is the exergy recovered, and the difference between the two is exergy destroyed. In the case of adiabatic compression, the useful work supplied constitutes exergy expended, and the increase in the exergy of the system constitutes the exergy recovered. Again, the difference between the two is the exergy destroyed. The ratio of the exergy increase of the system to the useful work input is the exergy efficiency of this compression process. We use the *exergy expended–recovered approach* in the development of the general exergy efficiency relation for closed systems since it is more appropriate for closed systems than the other approaches.

Consider a general closed system that may involve heat and work transfer into and out of the system as well as a change of state. The exergy change of a closed system of mass *m* during a process from state 1 to state 2 is given by Equation (10) as follows:

Δ*X*_sys_ = (*X*_2_
*− X*_1_)_sys_ = *m*[(*u*_2_
*− u*_1_) + *P*_0_(*v*_2_
*− v*_1_) − *T*_0_(*s*_2_
*− s*_1_) + (ke_2_ − ke_1_) + (pe_2_ − pe_1_)] (51)

For a closed system of mass *m* undergoing a process from state 1 to state 2, the reversible work relation, Equation (24), can be expressed as follows:

(52) $$W_{\mathrm{rev},\mathrm{out}}=(X_{Q\_in}-X_{Q\_out})+{\left(X_{1}-X_{2}\right)}_{\mathrm{sys}}=\sum \left(1-\frac{T_{0}}{T_{R}}\right)Q_{\mathrm{in}}+m(x_{nonflow,1}-x_{nonflow,2})$$

Or

(53) $$W_{\mathrm{rev},\mathrm{out}}=\sum \left(1-\frac{T_{0}}{T_{R}}\right)Q_{in}+m[\left(u_{1}-u_{2}\right)+P_{0}\left(v_{1}-v_{2}\right)-T_{0}\left(s_{1}-s_{2}\right)+\left({\mathrm{ke}}_{1}-{\mathrm{ke}}_{2}\right)+\left({\mathrm{pe}}_{1}-{\mathrm{pe}}_{2}\right)]$$

Negative values are used for *Q*_in_ for heat losses. A negative value for *W*_rev,out_ indicates reversible power input. For an *adiabatic* closed system that is *at rest* relative to the environment, the reversible work output becomes

(54) $$W_{rev,out,adiabatic}={\left(X_{1}-X_{2}\right)}_{\mathrm{sys}}=m[\left(u_{1}-u_{2}\right)+P_{0}\left(v_{1}-v_{2}\right)-T_{0}\left(s_{1}-s_{2}\right)]$$

The e*xergy change* of a closed system is treated as part of the exergy expended when it is negative and as part of the exergy recovered when it is positive. The exergy efficiency relations for the two cases are determined as follows:

(a) If Δ*X*_sys_ is positive (the system stores exergy),

*X*_expended_ = *X_W_*__in_
+ *X_Q_*__in_

*X*_recovered_ = *X_W_*__out_
+ *X_Q_*__out_ + (*X*_2_ − *X*_1_)_sys_

(55) $$\eta_{\mathrm{ex}}=\frac{X_{\mathrm{recovered}}}{X_{\mathrm{expended}}}=\frac{X_{W\_\mathrm{out}}+X_{Q\_\mathrm{out}}+{\left(X_{2}-X_{1}\right)}_{\mathrm{sys}}}{X_{W\_\mathrm{in}}+X_{Q\_\mathrm{in}}}=1-\frac{X_{\mathrm{destroyed}}}{X_{W\_\mathrm{in}}+X_{Q\_\mathrm{in}}}$$

(b) If Δ*X*_sys_ is negative (the system supplies exergy),

*X*_expended_ = *X_W__*_in_
+ *X_Q__*_in_ + (*X*_1_ − *X*_2_)_sys_

*X*_recovered_ = *X_W_*__out_
+ *X_Q_*__out_

(56) $$\eta_{\mathrm{ex}}=\frac{X_{\mathrm{recovered}}}{X_{\mathrm{expended}}}=\frac{X_{W\_\mathrm{out}}+X_{Q\_\mathrm{out}}}{X_{W\_\mathrm{in}}+X_{Q\_\mathrm{in}}+{\left(X_{1}-X_{2}\right)}_{\mathrm{sys}}}=1-\frac{X_{\mathrm{destroyed}}}{X_{W\_\mathrm{in}}+X_{Q\_\mathrm{in}}+{\left(X_{1}-X_{2}\right)}_{\mathrm{sys}}}$$

Here, for both cases,

(57) $$X_{\mathrm{destroyed}}=T_{0}S_{\mathrm{gen}}\;\mathrm{and}\;S_{\mathrm{gen}}=m\left(s_{2}-s_{1}\right)-\Sigma Q/T_{b}$$

Here, the exergy destroyed *X*_destroyed_ represents the exergy destroyed within the system boundaries. The closed system may involve more than one *W*_in_, *W*_out_, *Q*_in_, and *Q*_out_. If the heat transferred out of the system (*Q*_out_) consists of only heat loss to the environment (*Q*_out_ = *Q*_loss_), then we have $X_{Q\_\mathrm{out}}=X_{Q\_\mathrm{loss}}=Q_{\mathrm{loss}}\left(1-\frac{T_{0}}{T_{b}}\right),$ where *T_b_* is the average temperature of the system boundary.

When an *extended system* is considered in the analysis that includes the immediate surroundings of the device such that the system boundaries are at the environmental temperature of *T*_0_, the exergy transfer associated with heat loss becomes zero. Exergy efficiency in this case includes the exergy destroyed in the immediate surroundings as well as within the physical system. If the heat transferred out of the system (*Q*_out_) consists of only heat loss to the environment, the term *X_Q_*__out_ drops from the equations above since *X_Q_*__out_ = *X_Q_*__loss_ = 0 in this case.

Below, we give some case studies to demonstrate the determination of the exergy efficiency of closed systems by taking the environmental conditions to be *T*_0_ = 25 °C and *P*_0_ = 100 kPa.

**Figure 2:**
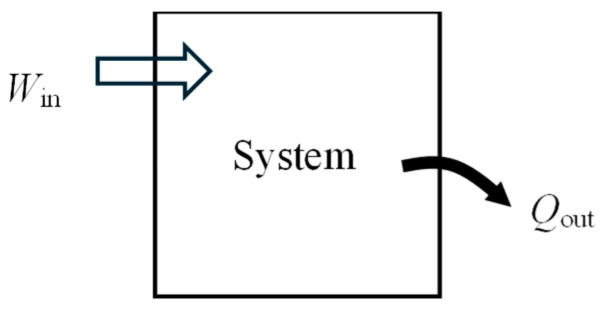
A rigid tank with heat and work interactions.

As a numerical application, consider a rigid tank filled with saturated water vapor with *m* = 0.5 kg and *T*_1_ = 120 °C (Figure 2). There is work input of *W*_in_ = 4100 kJ and some heat lost to the surroundings. The final pressure in the tank is *P*_2_ = 2 MPa. The exergy efficiency of this process is to be determined.

Exergy efficiency associated with a process is the exergy efficiency calculated for the extended system and it includes the exergy destruction that occurs within the immediate surroundings of the system. The energy balance for the rigid tank gives *Q*_out_ = 117 kJ. The exergy change of the system Δ*X*_sys_ is positive since there is net energy transfer to the system in the amount of 4100 − 117 = 3983 kJ. For the extended system, *X_Q_*__out_ = *X_Q_*__loss_ = 0 and we obtain *X*_expended_ = *X_W_*__in_ = 4100 kJ, *X*_recovered_ = *X_Q_*__out_ + (*X*_2_ − *X*_1_) = 0 + *X*_2_ − *X*_1_ = 3287 kJ, and *η*_ex_ = *X*_recovered_/*X*_expended_ = 0.802. That is, 80.2% of the exergy consumed is gained back during this process and the remaining 19.8% is wasted.

Using the value of exergy destroyed gives the same result for the extended system, as expected: entropy generation $S_{\mathrm{gen}}=m\left(s_{2}-s_{1}\right)+Q_{\mathrm{out}}/T_{0}$ = 2.73 kJ/kg·K, *X*_destroyed_ = *T*_0_*S*_gen_ = 813 kJ, and *η*_ex_ = 1 − *X*_destroyed_/*X*_expended_ = 0.802.

**Figure 3:**
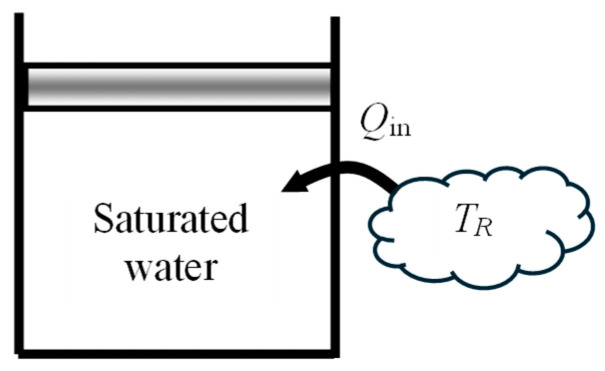
Heat transfer from a source to water in a piston–cylinder device.

As another numerical application, consider a piston–cylinder device filled with *m* = 0.5 kg of saturated liquid water at 200 kPa and heated at constant pressure by heat transferred from a heat reservoir at *T_R_* = 500 °C until the cylinder is filled with saturated vapor (Figure 3). Heat lost from the device is negligible. The exergy efficiency of this process is to be determined.

To capture the irreversibilities associated with heat transfer between the heat source and the piston–cylinder device, we consider an extended system whose boundary borders the heat source so that the temperature at that section of the boundary is the source temperature. The boundary work is determined to be *W_b_*_,out_ = 88.5 kJ and the useful work output is *X_W_*_,out_ = *W*_useful,out_ = *W_b_*_,out_ − *W*_surr_ = 88.5 − 44.2 = 44.3 kJ. The energy balance gives *Q*_in_ = 1101 kJ.

The exergy change of water is positive since Δ*X*_sys_ = *X*_2_ − *X*_1_ = 223 kJ is a positive quantity. Exergy expended is the exergy transferred from the heat source, which is the work potential of the heat transferred from the heat source, and exergy recovered is the increase in the exergy of the system plus the useful work output. The analysis gives *X*_expended_ = *X_Q_*__in_ = (1 − *T*_0_/*T_R_*)*Q*_in_ = 676 kJ, *X*_recovered_ = *X_W_*__out_ + (*X*_2_ − *X*_1_) = 44.3 + 223 = 267 kJ, and *η*_ex_ = *X*_recovered_/*X*_expended_ = 0.395. That is, 39.5% of the exergy consumed is gained back during this process and the remaining 60.5% is wasted. Alternately, using exergy destroyed, *X*_destroyed_ = *T*_0_*S*_gen_ = 410 kJ gives the same result for exergy efficiency for this process.

### 6.1. Exergy Efficiency Based on Reversible Work

It turns out that the relation *η*_ex_ = W_act,out_/W_rev,out_ (ratio of actual work output to reversible work output) is not suitable for the determination of exergy efficiency for closed systems. In the piston–cylinder application above, Equation (24) gives W_rev,out_ = 676 − 223 + 0 = 453 kJ. That is, when there are no irreversibilities, the piston–cylinder device analyzed here would produce 453 kJ of useful work instead of just 44.3 kJ when 1101 kJ of heat is transferred from the reservoir to the cylinder as water changes from saturated liquid to saturated vapor. If exergy efficiency is viewed as ratios of actual work to reversible work, this would give η_ex_ = W_act,out_/W_rev,out_ = 9.8%, which is considerably different from 39.5% obtained from the relation η_ex_ = X_recovered_/X_expended_.

It becomes interesting when the pressure of water in the cylinder is 100 kPa instead of 200 kPa and thus the useful work output is zero, *W*_useful,out_ = *W_b_*_,out_ − *W*_surr_ = 0. The two approaches in this case give *η*_ex_ = *X*_recovered_/*X*_expended_ = 32.6% and *η*_ex_ = *W*_act,out_/*W*_rev,out_ = 0%. The result obtained from the relation *η*_ex_ = *W*_act,out_/*W*_rev,out_ will always be zero regardless of other parameters of the problem as long as the pressure in the cylinder remains constant at 100 kPa during the process. The situation becomes even more interesting when the pressure is kept at 50 kPa which results in ‘negative’ actual work output, *W*_useful,out_ = − 81 kJ, and thus ‘negative’ exergy efficiency. But this cannot happen since the lowest value of exergy efficiency is zero, which happens when the entire exergy expended is wasted and thus the exergy recovered or output is zero.

This raises serious questions about the suitability of the relation *η*_ex_ = *W*_act,out_/*W*_rev,out_ for determining the exergy efficiency of piston–cylinder devices. For the 50 kPa pressure case, the correct result for exergy efficiency is *η*_ex_ = *X*_recovered_/*X*_expended_ = 184/708 = 25.9%. Using the exergy destroyed *X*_destroyed_ = *T*_0_*S*_gen_ = 525 kJ in calculations gives the same result.

### 6.2. Systems at Sub-Environmental Temperatures

Problems associated with systems or sources at temperatures below the environmental temperature can be challenging. Solving problems of this kind requires an intuitive understanding of the second-law concepts and critical thinking to be able to identify recovered and expended exergies properly.

**Figure 4:**
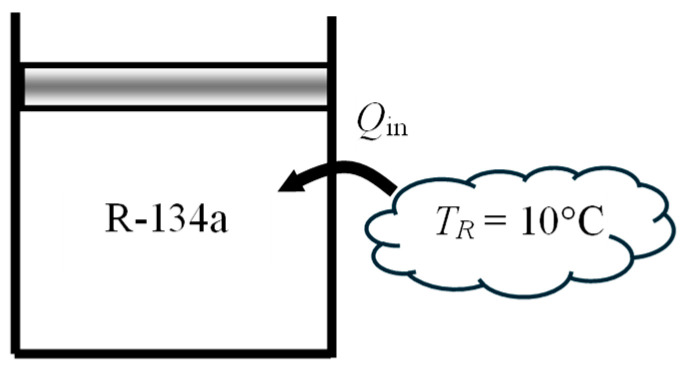
Heat transfer from a source to R-134a in a piston–cylinder device.

As a numerical application, consider a piston–cylinder device filled with *m* = 0.5 kg of saturated liquid refrigerant-134a at 160 kPa (*T*_sat_ = −15.6 °C) and heated at constant pressure by heat transferred from a heat reservoir at *T_R_* = 10 °C until the cylinder is filled with saturated vapor (Figure 4). Environmental conditions are again *T*_0_ = 25 °C and *P*_0_ = 100 kPa, and heat transfer with the environment is negligible. The exergy efficiency of this process is to be determined.

We observe that both the refrigerant temperature and source temperature are below the environmental temperature. This will offer a challenge when identifying the quantities that constitute exergy recovered and exergy expended. We also observe that, unlike water in the application above, the exergy of refrigerant-134 will decrease when heat is transferred to it since its temperature is below the environment temperature. The exergy of the source at 10 °C will increase as it transfers heat to the refrigerant at −15.6 °C since a heat engine operating between the environment at 25 °C and the source at 10 °C can now produce more work. Therefore, exergy expended during this process is the decrease in the exergy of the refrigerant, while exergy recovered is the increase in the exergy of the heat source plus the useful work output of the piston–cylinder device.

To properly account for the irreversibilities associated with heat transfer between the heat source and the piston–cylinder device, we consider an extended system whose boundary borders the heat source so that the temperature at that section of boundary is the source temperature. The boundary work is determined to be *W_b_*_,out_ = 9.8 kJ and the useful work output *X_W_*_,out_ = *W*_useful,out_ = *W_b_*_,out_ − *W*_surr_ = 9.8 − 6.1 = 3.7 kJ. The energy balance gives *Q*_in_ = 105 kJ. The exergy analysis gives *X*_expended_ = (*X*_1_ − *X*_2_) = 20.2 kJ, *X*_recovered_ = *X_W_*__out_ + *X_Q_*__in_ = 3.7 + 5.6 = 9.3 kJ, and *η*_ex_ = *X*_recovered_/*X*_expended_ = 0.459. That is, 45.9% of the exergy spent from the refrigerant is gained back during this process as work output and exergy storage in the heat source. The remaining 54.1% of the expended exergy is destroyed during this process. The alternate approach of using exergy destroyed *X*_destroyed_ = *T*_0_*S*_gen_ = 10.9 kJ gives the same result for exergy efficiency. This application also shows the utility and flexibility of the exergy efficiency definition *η*_ex_ = *X*_recovered_/*X*_expended_.

### 6.3. Resistance Heaters

Electric resistance heaters offer intriguing insight to exergy efficiency. The energy conversion efficiency of resistance heaters is always 100 percent since the entire electrical energy consumed is converted to thermal energy or heat. This gives the false impression that resistance heaters are perfect for heating from a thermodynamic point of view. But exergy analysis depicts a very different picture.

Consider a house maintained at a constant temperature of *T_H_* by resistance heaters when the cold outdoor temperature is *T*_0_. When there are no other heat sources in the house, energy balance reduces to ${\dot{Q}}_{\mathrm{loss}}={\dot{W}}_{\mathrm{in}}$ since the heat loss from the house must be made up by the heater for the indoor temperature to remain constant. Exergy input or exergy expended for this heating process is electric power input. Exergy recovered is the exergy content of the heat loss, which is the work that can be produced by a reversible heat engine operating between the temperature limits of *T_H_* and *T*_0_. Considering that ${\dot{Q}}_{\mathrm{loss}}={\dot{W}}_{\mathrm{in}}$, the exergy efficiency becomes

$$\eta_{\mathrm{ex}}=\frac{{\dot{X}}_{\mathrm{recovered}}}{{\dot{X}}_{\mathrm{expended}}}=\frac{{\dot{Q}}_{\mathrm{loss}}\left(1-T_{0}/T_{H}\right)}{{\dot{W}}_{\mathrm{in}}}=1-\frac{T_{0}}{T_{H}}$$

which is the thermal efficiency of a Carnot heat engine that operates between the temperature limits of *T_H_* and *T*_0_, as expected. For an extended system that includes the house and its immediate surroundings so that the boundary temperature is *T*_0_, there is no exergy recovered and thus the exergy efficiency is zero. This is a more realistic scenario since it is not practical to make use of the heat lost from a building. For the same considerations, the exergy efficiency of electric radiant heaters used outdoors is also zero.

**Figure 5:**
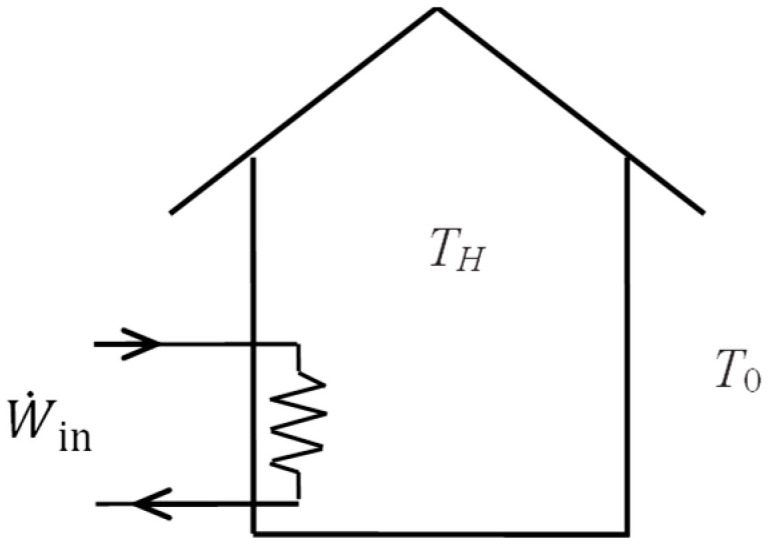
A house heated by resistance heaters.

As a numerical application, consider a house heated by a 20 kW electric resistance heater that keeps the house at *T_H_* = 25 °C when the outdoor temperature is *T*_0_ = 10 °C (Figure 5). Taking the house as the system, the exergy efficiency of this heating process is to be determined.

The exergy efficiency is determined to be *η*_ex_ = 1 − *T*_0_/*T_H_* = 1 − 283/298 = 0.050 or 5 percent. The low value of exergy efficiency demonstrates the high degree of irreversibility associated with resistance heating. Of the 20 kW exergy expended, only 1.0 kW of exergy is recovered while the remaining 19 kW of exergy is destroyed, which can also be calculated from ${\dot{X}}_{\mathrm{dest}}=T_{0}{\dot{S}}_{\mathrm{gen}}=T_{0}{\dot{Q}}_{\mathrm{loss}}/T_{H}$. Exergy efficiency can also be determined from $\eta_{ex}=1-{\dot{X}}_{\mathrm{destroyed}}/{\dot{X}}_{\mathrm{expended}}\;=1-19/20\;=\;0.05$.

## 7. Exergy Efficiency of Unsteady-Flow Systems

Unsteady-flow systems usually involve transient operations or batch operations such as charging and discharging processes. The analysis of unsteady-flow systems is similar to the analysis of closed systems, except that the unsteady-flow systems involve mass flow into or out of the system. Therefore, exergy efficiency relations for unsteady-flow systems can be obtained from closed-system relations by incorporating exergy flow terms with mass. Unlike steady-flow systems, unsteady-flow systems involve changes with time within the system boundaries during the process. Therefore, we use the *exergy expended–recovered approach* here as we did for closed systems.

The change in the exergy content of the system is the difference between the exergies in the final and initial states, from Equation (8), as follows:

(58) $$X_{\mathrm{initial}}=X_{1}=m_{1}[\left(u_{1}-u_{0}\right)+P_{o}\left(v_{1}-v_{0}\right)-T_{0}\left(s_{1}-s_{0}\right)+{\mathrm{ke}}_{1}+{\mathrm{pe}}_{1}]$$

(59) $$X_{\mathrm{final}}=X_{2}=m_{2}[\left(u_{2}-u_{0}\right)+P_{o}\left(v_{2}-v_{0}\right)-T_{0}\left(s_{2}-s_{0}\right)+{\mathrm{ke}}_{2}+{\mathrm{pe}}_{2}]$$

(60) $$∆X_{\mathrm{sys}}=X_{\mathrm{final}}-X_{\mathrm{initial}}={\left(X_{2}-X_{1}\right)}_{\mathrm{sys}}$$

Assuming uniform flow, exergy transfers by mass entering and leaving are, from Equation (9):

(61) $$X_{\mathrm{mass},\mathrm{in}}=m_{in}x_{\mathrm{flow},\mathrm{in}}=m_{in}[\left(h_{in}-h_{0}\right)-T_{0}\left(s_{in}-s_{0}\right)+{\mathrm{ke}}_{in}+{\mathrm{pe}}_{in}]$$

(62) $$X_{\mathrm{mass},\mathrm{out}}=m_{out}x_{\mathrm{flow},\mathrm{out}}=m_{out}[\left(h_{out}-h_{0}\right)-T_{0}\left(s_{out}-s_{0}\right)+{\mathrm{ke}}_{out}+{\mathrm{pe}}_{out}]$$

Here, *m*_in_ is the amount of mass entering, *m*_out_ is the amount of mass leaving, *m*_1_ is the initial mass, and *m*_2_ is the final mass in the system. If the flow is not uniform, exergy transfers by mass should be determined via integration as $X_{\mathrm{mass},\mathrm{in}}=\int x_{\mathrm{flow},\mathrm{in}}\delta m_{in}$ and $X_{\mathrm{mass},\mathrm{out}}=\int x_{\mathrm{flow},\mathrm{out}}\delta m_{out}$.

The exergy change of the system Δ*X*_sys_ is treated as part of the exergy expended when it is negative and as part of the exergy recovered when it is positive:

(a) If Δ*X*_sys_ is positive,

*X*_expended_ = *X_W_*__in_
+ *X_Q_*__in_+ *X*_mass,in_ (63)

(64) $$X_{\mathrm{recovered}}=X_{W\_\mathrm{out}}+X_{Q\_\mathrm{out}}+X_{\mathrm{mass},\mathrm{out}}+{\left(X_{2}-X_{1}\right)}_{\mathrm{sys}}$$

(65) $$\begin{array}{cc} \eta_{\mathrm{ex}}=\frac{X_{\mathrm{recovered}}}{X_{\mathrm{expended}}} & =\frac{X_{W\_\mathrm{out}}+X_{Q\_\mathrm{out}}+X_{mass,out}+{\left(X_{2}-X_{1}\right)}_{\mathrm{sys}}}{X_{W\_\mathrm{in}}+X_{Q\_\mathrm{in}}+X_{mass,in}}\\ & =1-\frac{X_{\mathrm{destroyed}}}{X_{W\_\mathrm{in}}+X_{Q\_\mathrm{in}}+X_{mass,in}} \end{array}$$

(b) If Δ*X*_sys_ is negative,

*X*_expended_ = *X_W_*__in_
+ *X_Q_*__in_ + *X*_mass,in_ + (*X*_1_
− *X*_2_)_sys_ (66)

*X*_recovered_ = *X_W_*__out_
+ *X_Q_*__out_ + *X*_mass,out_ (67)

(68) $$\begin{array}{cc} \eta_{\mathrm{ex}}=\frac{X_{\mathrm{recovered}}}{X_{\mathrm{expended}}} & =\frac{X_{W_{\mathrm{out}}}+X_{Q_{\mathrm{out}}}+X_{mass,out}}{X_{W_{\mathrm{in}}}+X_{Q_{\mathrm{in}}}+X_{mass,in}+{\left(X_{1}-X_{2}\right)}_{\mathrm{sys}}}\\ & =1-\frac{X_{\mathrm{destroyed}}}{X_{W\_\mathrm{in}}+X_{Q\_\mathrm{in}}+X_{mass,in}+{\left(X_{1}-X_{2}\right)}_{\mathrm{sys}}} \end{array}$$

where

$$X_{\mathrm{destroyed}}=T_{0}S_{\mathrm{gen}}$$

and

(69) $$S_{\mathrm{gen}}=(m_{2}s_{2}-m_{1}s_{1})+(m_{out}s_{out}-m_{in}s_{in}+Q_{in}/T_{b,in}-Q_{out}/T_{b,out})$$

When an *extended system* is considered in an analysis that includes the immediate surroundings of the device, the exergy associated with heat loss becomes zero. Exergy efficiency in this case includes the exergy destroyed in the immediate surroundings as well as within the physical system. If the analysis is to be limited to the system only, then the exergy associated with heat loss should be considered by including the term ${\dot{X}}_{Q\_\mathrm{loss}}={\dot{Q}}_{\mathrm{loss}}(1-T_{0}/T_{b})$ in the recovered exergy expression. Here, *T_b_* is the average temperature of the system boundary.

When kinetic and potential energy changes associated with the system and the mass streams are negligible, mass and energy balances for an unsteady-flow system become

(70) $$m_{\mathrm{in}}-m_{\mathrm{out}}=m_{2}-m_{1}$$

(71) $$W_{\mathrm{in}}+Q_{\mathrm{in}}+m_{\mathrm{in}}h_{\mathrm{in}}=W_{\mathrm{out}}+Q_{\mathrm{out}}+m_{\mathrm{out}}h_{\mathrm{out}}+{(m}_{2}u_{2}-m_{1}u_{1}).$$

The reversible work relation in Equation (24) can be expressed in this case as

(72) $$W_{\mathrm{rev},\mathrm{out}}=\left(X_{Q\_in}-X_{Q\_out}\right)+(X_{mass,in}-X_{mass,out})+{\left(X_{1}-X_{2}\right)}_{\mathrm{sys}}$$

or

(73) $$W_{\mathrm{rev},\mathrm{out}}=\sum \left(1-\frac{T_{0}}{T_{R}}\right)Q_{\mathrm{in}}+\left(m_{in}x_{flow,in}-m_{out}x_{flow,out}\right)+\left(m_{1}x_{nonflow,1}-m_{2}x_{nonflow,2}\right)$$

Negative values are used for *Q*_in_ for heat losses. A negative value for *W*_rev,out_ indicates reversible power input.

**Figure 6:**
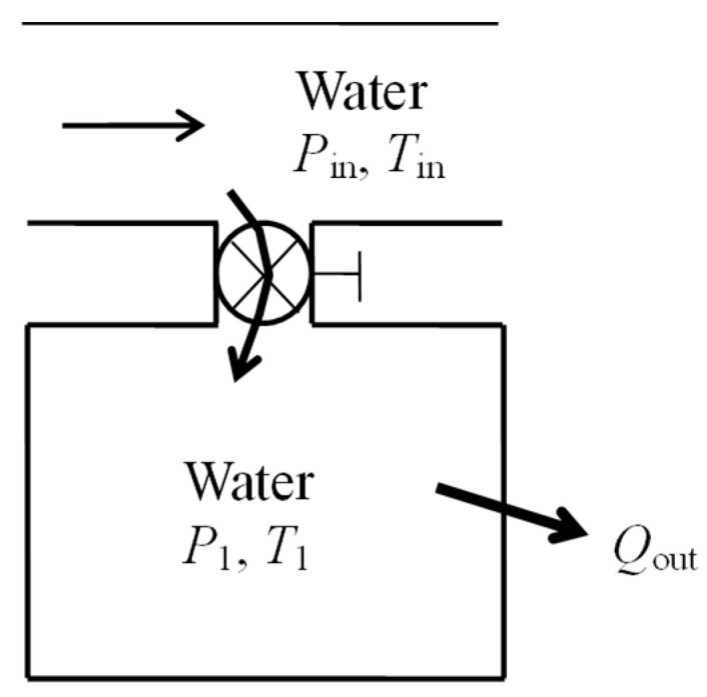
A rigid water tank being filled with steam from a supply line.

As a numerical application, we consider a 0.5 m^3^ rigid tank that initially contains water at *T*_1_ = 200 °C and *P*_1_ = 500 kPa (Figure 6). Steam at *T*_in_ = 500 °C and *P*_in_ = 2000 kPa is allowed to enter the tank through a supply line until the tank reaches a final state *T*_2_ = 300 °C and *P*_2_ = 800 kPa. Heat is lost from the tank to the surroundings during the process. The exergy efficiency of the extended system that includes the tank and its immediate surroundings is to be determined.

We take the rigid tank and its immediate surroundings as the extended system. The mass and energy balances give *Q*_out_ = 64.1 kJ for heat loss. The exergy change of the system Δ*X*_sys_ is positive since *X*_2_ > *X*_1_. Using the relations above, we obtain *X*_expended_ = 460 kJ, *X*_recovered_ = 355 kJ, and *η*_ex_ = *X*_recovered_/*X*_expended_ = 0.772. Therefore, 77.2% of the exergy consumed is recovered during this process and the remaining 22.8% is wasted. The alternative relation *η*_ex_ = 1 − *X*_destroyed_/*X*_expended_ with *S*_gen_ = 0.346 kJ/kg·K and *X*_destroyed_ = *T*_0_*S*_gen_ = 104 kJ also gives the same result for exergy efficiency.

## 8. Conclusions

In this paper, general exergy efficiency relations are developed for closed and unsteady-flow systems. Also, the merits of different definitions of exergy efficiency are assessed, and the concepts of exergy loss and exergy destruction are put into proper perspectives.

*Exergy efficiency* is a measure of the actual performance of an energy system relative to the thermodynamically best possible performance, which occurs under reversible conditions characterized by zero entropy generation or zero exergy destruction. Exergy efficiency is a measure of thermodynamics perfection, varying from 100 percent for a reversible (thermodynamically perfect) process or device to zero when the exergy consumed or expended is completely wasted. Exergy efficiency is commonly expressed as the ratio of the exergy output *X*_out_ to the exergy input *X*_in_ as *η*_ex_ = *X*_out_/*X*_in_. But this definition is best suited for steady-flow systems. For closed and unsteady-flow systems, the more appropriate definition is *η*_ex_ = *X*_recovered_/*X*_expended_ = 1 − *X*_destroyed_/*X*_expended_ since it properly accounts for the exergy change of the systems.

Exergy efficiency relations for general closed and unsteady-flow systems are developed using this definition and the results are given by Equations (55), (56), (65), and (68). The exergy efficiency relations *η*_ex_ = *W*_act,out_/*W*_rev,out_ and *η*_ex_ = *W*_rev,in_/*W*_act,in_ are appropriate for adiabatic or nonadiabatic steady-flow devices that are intended to produce work or require work input, respectively, but not so for other devices. For a chemically reacting steady-flow system with all reactants entering and products leaving at the standard reference temperature *T*_0_ and pressure *P*_0_, the reversible work is expressed by Equation (45).

Both *exergy destruction* and *exergy lost* represent waste of exergy. A wasted exergy quantity is regarded either as exergy loss or exergy destruction, depending on the choice of system boundary. But this division appears to be superficial since exergy loss associated with heat loss or exhausted substances are treated as *exergy loss* if the physical device is taken as the system, and as *exergy destruction* if the extended system is taken as the system. When determining the exergy efficiency of the physical system, the simplest approach is to consider all exergy leaving physical system as *exergy output* or *exergy recovered* and not deal with *exergy loss* at all. The exergy efficiency determined this way is equivalent to *η*_ex_ = 1 − *X*_destroyed_/*X*_expended_ where *X*_destroyed_ is the exergy destruction within the boundaries of the physical system. The sum of the exergy destruction within the physical system and that of the immediate surroundings gives the total exergy destruction associated with the process. The fraction of exergy destruction due to exergy loss can be determined easily if desired by dividing it by the exergy expended.

Considering that exergy loss is eventually destroyed in the immediate surroundings, it makes practical sense to work with the *extended system* and treat both quantities as exergy destruction to avoid confusion. Analysis is simplified when the extended system is used since exergy loss, which is the exergy output associated with heat loss and purged substances, if any, becomes zero in that case, and thus the need to evaluate it at the physical system boundary is eliminated. When the extended system is analyzed, the *exergy input–output* and *exergy expended–recovered* formulations give the same result for exergy efficiency.

The proper choice of the system may result in obtaining a more meaningful value for exergy efficiency. Taking the *physical system* as the system and treating exergy loss as part of the recovered exergy results in higher exergy efficiency. But this gives a false impression of better performance since this exergy is eventually destroyed in the immediate surroundings. Therefore, taking an *extended system* that includes the device and its immediate surroundings where temperature and concentration gradients occur results in a more realistic value for exergy efficiency. The exergy efficiency of the extended system includes exergy destroyed within the immediate surroundings as well as within the physical system, and thus it gives a more conservative result. The difference between the exergy efficiency of the physical system and that of the extended system gives the efficiency loss due to external irreversibilities that occur in the immediate surroundings. In the end, the calculated exergy efficiency should make the most sense and provide the best insight when judging the performance of the system.

## Data Availability

Data is contained within the article.

## Nomenclature

COP	coefficient of performance
*e*	specific energy (kJ/kg)
*g*	gravitational acceleration (m^2^/s)
*G*	Gibbs function (kJ)
*h*	enthalpy (kJ/kg)
ke	kinetic energy (kJ/kg)
*m*	mass (kg)
*N*	mole number (kmol)
*P*	pressure (kPa)
pe	potential energy (kJ/kg)
*Q*	heat transfer (kJ)
*R*	gas constant (kJ/kg·K)
*s*	entropy (kJ/kg·K)
*T*	temperature (K)
*u*	internal energy (kJ/kg)
*v*	specific volume (m^3^/kg)
*V*	velocity (m/s)
***V***	volume (m^3^)
*W*	work (kJ)
*X*	exergy (kJ)
*y*	mole fraction
*z*	elevation (m)
Greek letters	
*η*	efficiency
*μ*	chemical potential (kJ/kg)
*ρ*	density (kg/m^3^)
Subscripts	
0	environmental state
act	actual
*b*	boundary
ch	chemical
env	environment
ex	exergy
gen	generation
*H*	high temperature
*i*	component *i*
in	input
*m*	mixture
*L*	low temperature
*p*	product
out	output
*r*	reactant
*R*	reservoir
rev	reversible
surr	surroundings
sys	system
Superscipts	
^˙^	time rate
¯	per unit mole
^0^	standard reference state
